# Anxiolytic Effects of Flavonoids in Animal Models of Posttraumatic Stress Disorder

**DOI:** 10.1155/2012/623753

**Published:** 2012-12-13

**Authors:** Li-Ming Zhang, Jia-Zhi Yao, Yang Li, Kai Li, Hong-Xia Chen, You-Zhi Zhang, Yun-Feng Li

**Affiliations:** ^1^Department of New Drug Evaluation, Beijing Institute of Pharmacology and Toxicology, Beijing 100850, China; ^2^The 4th Ward of Psychiatry Department, The 261th Hospital of the People's Liberation Army, Beijing 100094, China; ^3^Clinical Laboratory, The 261th Hospital of the People's Liberation Army, Beijing 100094, China

## Abstract

The dysregulation of the serotonergic system has long been recognized as an important factor underlying the pathophysiology of PTSD. To date, SSRIs have already been established as the firstline pharmacotherapeutic agents for treating acute and chronic PTSD. However, SSRIs largely have several disadvantages which limit their utility. Our previous study has also shown that administration of the total flavonoids, isolated from the extract of Xiaobuxin-Tang (XBXT, mild mind-easing decoction), comprising four Chinese medicines including Haematitum, Flos Inulae, Folium Phyllostachydis Henonis, and Semen Sojae Preparatum, exerted significant antidepressant-like effect in chronically mildly stressed rats, possibly mediated by serotonergic activation. Since the central serotonergic dysfunction is an important and well-known cause mediating the pathophysiology of trauma-related symptoms in PTSD, it is reasonable to predict that flavonoids may exert therapeutic effects on PTSD in animal models. Therefore, the present study aims to examine the effect of flavonoids in alleviating the enhanced anxiety and fear response induced in two PTSD animal models. Ser, an SSRI, was administered as a positive control. Furthermore, the changes of brain monoaminergic neurotransmitters after chronic flavonoids administration have also been assessed in SPS-treated rats.

## 1. Introduction

PTSD is a severe, disabling anxiety disorder that may occur after exposure to severely traumatic event. According to the DSM-IV diagnostic criteria, PTSD involves characteristic features such as persistently reexperienced trauma, avoidance, numbing, and hyperarousal. As a complex disorder, to date, the exact aetiology of PTSD is unclear, and research into the underlying neurobiology has implicated the alterations of a myriad of neurotransmitter and neuroendocrine systems, including serotonin, norepinephrine, and GABA, as well as dysregulation of the hypothalamic-pituitary-adrenal axis and the locus coeruleus-norepinephrine system [1–4]. Among them, the dysregulation of the serotonergic system has long been recognized as an important factor underlying the pathophysiology of PTSD. Many evidence—including decreased serum concentrations of serotonin (5-HT), decreased density of platelet 5-HT uptake sites, and a blunted prolactin response to D-fenfluramine (indicative of central 5-HT hypoactivity)—suggests that 5HT activity is decreased in PTSD patients [5–10]. Perhaps the best grounds for proposing that a 5-HT dysfunction exists in PTSD resides in the beneficial treatment effects of SSRIs, which, to date, have already been established as the first-line pharmacotherapeutic agents for treating acute and chronic PTSD. However, SSRIs largely have disadvantages including delayed onset of action, partial response with residual symptoms or non-response, and severe side effects (e.g., loss of sexual drive, gastrointestinal effects, changes in body weight), which limit their utility and indicate a major unmet medical need to explore more promising treatment approaches in PTSD [11–17]. 

Traditional Chinese medicine (TCM), because of its better compliance and lower side effects [18], draws more and more attentions and provides a prospective alternative to the treatment of several mental disorders [18–22]. Xiaobuxin-Tang (XBXT, mild mind-easing decoction), comprising 4 Chinese medicines including Haematitum, Flos Inulae, Folium Phyllostachydis Henonis, and Semen Sojae Preparatum, was originally recorded in the silk scroll manuscript of “Fuxinjue Zangfu Yongyao Fayao” (The essentials of medication for viscera and organs, aimed at Taoist practice), written one thousand years ago and discovered in Mogao Caves of Dunhuang. The three ingredients of XBXT, Flos Inulae, Folium Phyllostachydis Henonis, and Semen Sojae Preparatum, all contain a large amount of flavonoids, and during the process of extraction, the flavonoids may react with Haematitum, the fourth ingredient of XBXT, to form some flavone complexes. *In vivo* studies show that flavonoids can be absorbed after oral administration, pass the blood-brain barrier, and exert various effects on the CNS. Several lines of evidence have demonstrated the effects of flavonoids on memory, cognition, and neurodegeneration [23, 24]. 

Our previous study has also shown that administration of the total flavonoids, isolated from the extract of XBXT, exerted significant antidepressant-like effect in chronically mildly stressed rats, possibly mediated by serotonergic activation [25–27]. Since the central serotonergic dysfunction is an important and well-known cause mediating the pathophysiology of trauma-related symptoms in PTSD, it is reasonable to predict that flavonoids may exert therapeutic effects on PTSD in animal models. Therefore, the present study aims to examine the effect of flavonoids in alleviating the enhanced anxiety and fear response induced in two PTSD animal models. Ser, an SSRI, was administered as a positive control. Furthermore, the changes of brain monoaminergic neurotransmitters after chronic flavonoids administration have also been assessed in SPS-treated rats.

## 2. Materials and Methods

### 2.1. Animals

Both male ICR mice (18 ± 2 g) and male Sprague-Dawley rats (180 ± 10 g) were purchased from Beijing Vital Laboratory Animal Technology Company (Beijing, China). Animals were maintained under standard conditions of controlled temperature (23 ± 1°C), humidity (45%), and lighting (12 h/d). Experiments were conducted according to the National Institute of Health Guide for the Care and Use of Laboratory Animals [28]. The experimental procedures were approved by the institutional committee on animal care and use, and all efforts were made to minimize animal suffering and reduce the number of animals used for the experiments. 

### 2.2. Drugs and Drug Administration

The sertraline was purchased from Sigma-Aldrich (St. Louis, MO, USA). 

Traditional Chinese medicines Haematitum, Flos Inulae, Folium Phyllostachydis Henonis, and Semen Sojae Preparatum were purchased from Beijing Tongrentang Drugstore (Beijing, China) and were identified by Prof. Lian-Sheng Shen (School of Chinese Medicine, Beijing University of Chinese Medicine) as calcined product of ochery hematite, flowers of *Inula japonica *Thunb. leafs of *Phyllostachys nigra* (Lodd.) Munro var.* henonis* (Mitf.) Stapf ex Rendle, fermented product of *Glycine max (L.) *Merr, respectively. The voucher specimens (nos. 02002, 02003, 02004, and 02005, resp.) were deposited in the Laboratory of Phytochemistry, Beijing Institute of Pharmacology and Toxicology, China. The extraction of flavonoids was carried out according to the method of An et al. [27]. Using lutin as standard substance, the content of flavones in the extract was determined as 76.03% by colorimetric method. 

Ser or flavonoids was dissolved in saline and administered by intragastric gavage (i.g.) in a volume of 20 mL/kg (mice) or 2 mL/kg (rats).

### 2.3. Long-Term Behavioral Effects of Flavonoids after Electric Foot-Shocks Procedures

The experimental procedure was carried out as described previously [29]. For the training session, a plexiglass chamber (20 × 10 × 10 cm) with stainless steel grid floor (9 mm interval) was used. Electric foot-shocks were delivered through the grid floor by an isolated shock generator (Med Associates Inc., USA). Each mouse was placed in the chamber, and after a 5-min adaptation period, a total of 15 intermittent inescapable foot-shocks (intensity: 0.8 mA, interval: 10 s, and duration: 10 s) were delivered for 5 min. Control animals were placed in the same chamber for 10 min, without electric foot-shocks. From the first day (day 1) after the foot-shock procedure, Ser (15 mg/kg) or flavonoids (12.5, 25, and 50 mg/kg, resp.) was given by intragastric gavage (i.g.) once a day at 8:00-9:00 a.m. The drug doses and the administration time were selected according to our previous studies. 

#### 2.3.1. Contextual Freezing Measurement

All animals were exposed to the reminder situation, that is, the same chamber where the foot-shocks had been delivered, but with no further foot-shocks, for 5 min on day 3, 8, and 15, respectively. Freezing behavior, defined as an absence of all movement (except for respiration), was scored during the situational reminder. Total cumulative freezing time (total seconds spent freezing during each assessment period) was measured and scored by a trained observer blind to the treatment conditions [30].

#### 2.3.2. The Staircase Test in Mice

The staircase was made from polyvinylchloride and consisted of 5 identical steps (2.5 cm high, 10 cm wide, and 7.5 cm deep). The height of the walls was constant (12.5 cm above the stairs) along the entire length of the staircase. On day 18 after foot-shocks, the mouse was placed on the floor of the box with its back toward the staircase. Each mouse was placed individually onto the staircase. During a 3-min period, number of rearing-effect and number of steps climbed were recorded. A step was considered climbed only if the mouse placed all 4 paws on the stair. The number of steps descended was not counted. At the end of 3 min, the mouse was removed, and the staircase was cleaned with an alcohol sponge to eliminate any residual odors. The treatments were randomized, and the observer was blind to the grouping. All studies were carried out between 8:00–11:00 a.m.

### 2.4. Long-Term Behavioral Effects of Flavonoids in Rats after Exposure to SPS

The SPS procedure was performed as described previously [31, 32]. Briefly, after a 2-week acclimatization period, the rats were restrained for 2 h, and then each rat was immobilized inside a disposable clear polyethylene rodent restraint cone. The large end of the cone was closed with tape. The bag size was adjusted according to the size of the animal to achieve a complete immobilization. A hole in the small end of the cone allowed the rats to breathe freely. After that, rats were individually placed in a clear acrylic cylinder (24-cm diameter, 50 cm height), filled with water (24°C) to 2/3 of its height, and forced to swim for 20 min. Following 15 min recuperation, rats were then exposed to diethyl ether until loss of consciousness. Control rats were kept in a room adjacent to the SPS rats during the treatment and handled twice for several minutes each time. From the first day (day 1) after the SPS procedure, Ser (15 mg/kg) or flavonoids (12.5, 25, and 50 mg/kg, resp.) was given by p.o. once per day at 8:00-9:00 a.m.

#### 2.4.1. Locomotor Activity Test

Thirteen days after SPS, the locomotor activity was measured by placing each rat individually in a clear open field (36 × 36 × 36 cm) with black rubber floor. The animal was allowed to habituate to the environment for 5 min; the spontaneous movement comprising the traveling distance and time of ambulation was automatically recorded by VIDEO-MEX-V image analytic system (Columbus Instruments, USA) in the subsequent 5 min. 

#### 2.4.2. Contextual Fear Paradigm

The contextual fear paradigm was conducted after the end of the 14-day drug treatments. On the first day (day 14 after SPS), each rat was exposed to the conditioning context (180 s, in the conditioning chamber (60 × 21 × 30 cm) without any stimulation). Immediately after that, a foot-shock (0.8 mA, 4 s) through a stainless steel grid floor (Med Associates Inc. USA) was given. Twenty-four hours after the initial foot-shock (day 15 after SPS), the rat was placed in the same conditioning chamber where the previous foot-shocks were conducted, and the contextual fear response was then evaluated by measuring the duration of freezing behavior in a 5 min interval. “Freezing” behavior was defined as a total absence of body or head movement except for that associated with breathing which was rated by the observers who were blind to the grouping, and the data were recorded (in seconds) as the average time spent freezing [30]. 

#### 2.4.3. The Elevated Plus Maze (EPM) Test

This paradigm was well validated in detecting responses to external stressful stimuli. The apparatus consisted of 4 branching arms (50 × 10 cm) with 2 arms open and the other 2 closed with dark walls (14 cm high). The arms were connected by a center platform (10 × 10 cm), and the maze was 50 cm above the ground. Eighteen days after SPS (day 18), individual rat was placed in the central platform, facing the closed arms. For the purpose of analysis, open-arm activity was quantified as the time spent on the open arms relative to the total time spent in both arms (open/total × 100), and the number of entries into open arms relative to the total number of entries into any arm (open/total × 100). Rats were scored as entering an open or closed arm only when all 4 paws passed over the dividing line. The maze was cleaned with a 5% ethanol/water solution after each test to remove any confounding olfactory cues and dried thoroughly between sessions. 

#### 2.4.4. Long-Term Effect of Flavonoids on Monoamine Neurotransmitters after Exposure to SPS

Rats used in the EPM test were sacrificed by decapitation, and the hippocampus and prefrontal cortex were rapidly removed. The tissues were weighed and sonicated in 0.4 M HClO_4_ containing 0.5 mM Na_2_-EDTA and 0.01% L-cys cocktail solution on ice. The homogenates were then centrifuged at 12,000 rpm, 4°C for 30 min. Supernatant was collected and stored at −80°C until further use.

The high-performance liquid chromatography (HPLC) system consisted of a microbore reverse-phase column (particle size 5 *μ*m, 150 × 4.6 mm; Model C-18, DIKMA Technologies Ltd., Beijing, China), an Agilent 1100 pump (flow rate 1.0 mL/min; Agilent Technologies, Palo Alto, CA, USA), and a Hewlett-Packard HP 1049A glassy carbon amperometric detector (Agilent Technologies, Palo Alto, CA, USA). Concentrations of dopamine (DA), 5-HT, and norepinephrine (NE) in hippocampus and cortex were simultaneously detected. The mobile phase composed of 85 mM citrate, 100 mM sodium acetate, 0.9 mM octyl-sodium sulfate, 0.2 mM EDTA, and 15% HPLC grade methanol, pH 3.7. External standard curves were used to quantify the amounts of NE, 5-HT, and DA in each sample by calculating the area under curve (AUC). The volume of injection was 50 *μ*L. The detection limit of the assay was 20 pg/sample.

### 2.5. Statistical Analysis

All data were expressed as mean ± SEM. The significance of difference between treatment and control groups was determined by One Way Analysis of Variance (ANOVA) followed by Dunnett's *t*-test. For all tests, differences with *P* < 0.05 were considered significant.

## 3. Results

### 3.1. Long-Term Behavioral Effects of Flavonoids after Electric Foot Shocks in Mice

**Figure 1:**
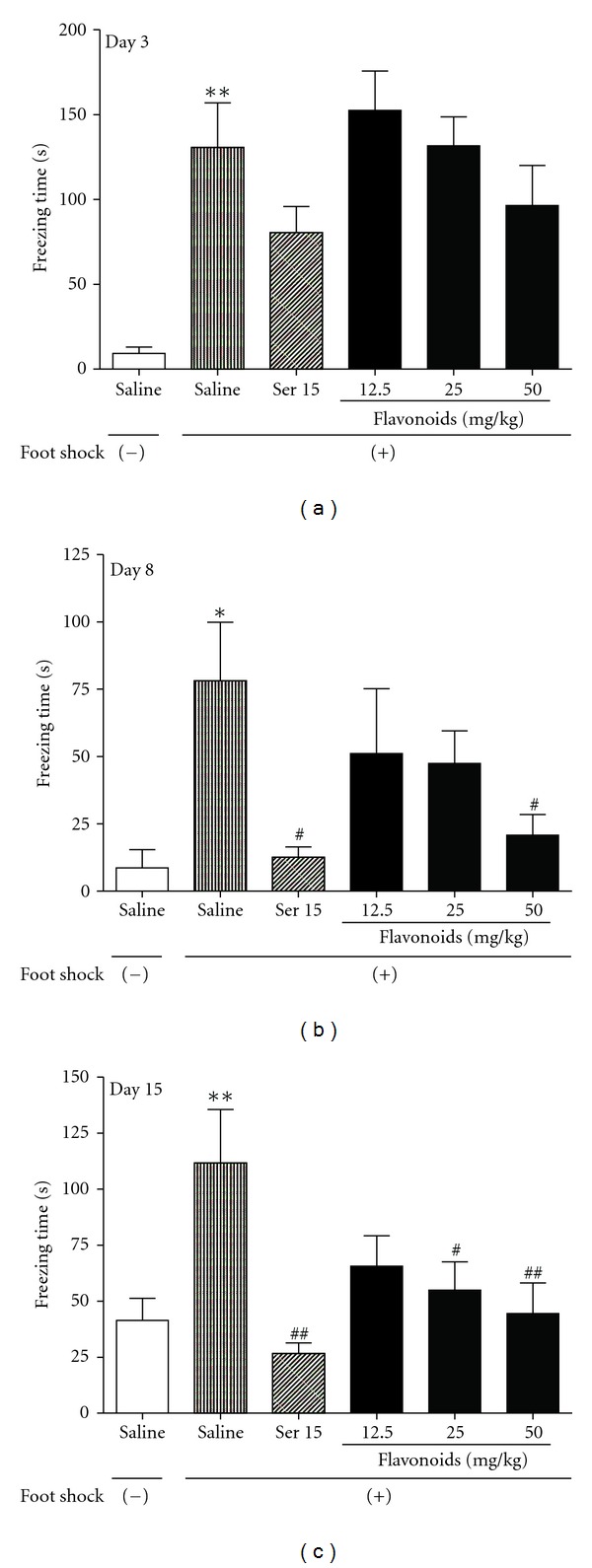
The effects of repeated treatment with Ser or flavonoids on freezing behavior in mice after exposure to electric foot-shocks. The total cumulative freezing time was determined on day 3, 8, and 15. Daily administrations of Ser or flavonoids were started from the first day of training session. Data are presented as mean ± SEM (*n* = 10). *P < 0.05, **P < 0.01 compared with foot shock (−) group; ^#^ *P* < 0.05, ^##^ *P* < 0.01 compared with saline-treated foot shock (+) group (ANOVA followed by Dunnett's *t*-test).

Exposure to foot shock significantly increased the contextual freezing response (day 3: *F*[5, 54] = 6.833, *P* < 0.01; day 8: *F*[5, 54] = 3.222, *P* < 0.05; and day 15: *F*[5, 54] = 4.303, *P* < 0.01). Repeated treatment with Ser for 15 days significantly reduced the freezing behavior (*P* < 0.01) induced by foot shock. A similar effect was also observed after repeated administration of flavonoids at doses of 25 and 50 mg/kg (*P* < 0.05 and *P* < 0.01 resp.; Figure 1).

**Figure 2:**
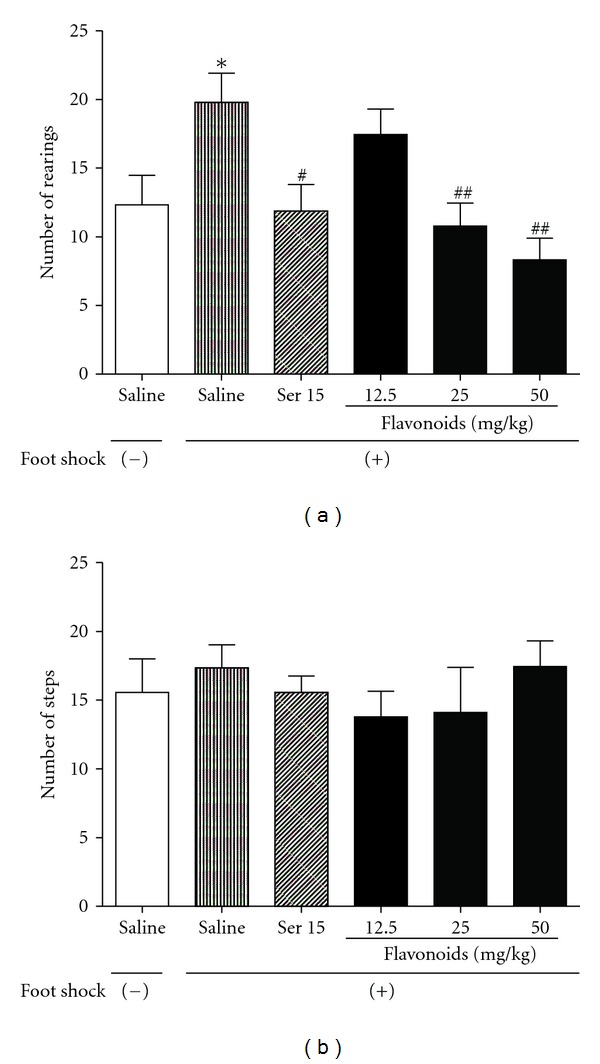
The effects of repeated treatment with Ser or flavonoids on the behavior of mice in the staircase test. (a) number of rearings and (b) number of steps climbed by mice during a 3-min period. Data are presented as mean ± SEM (*n* = 10). *P < 0.05 compared with foot-shocks. (−) group; ^#^ *P* < 0.05, ^##^ *P* < 0.01 compared with saline-treated foot-shocks (+) group (ANOVA followed by Dunnett's *t*-test).

In the staircase test, mice that had been previously exposed to foot shocks and situational reminders exhibited an increased number of rearings (*F*[5, 54] = 5.163, *P* < 0.05) but failed to demonstrate a significant change in number of steps (*F*[5, 54] = 0.5102, *P* > 0.05). These results indicate that the animals still avoided the aversive-like compartment and that they exhibited a fear response to the context associating with traumatic events. ANOVA showed that repeated administrations of flavonoids (25 and 50 mg/kg, *P* < 0.01) or Ser (15 mg/kg, *P* < 0.01) significantly improved the behavioral deficits induced by the aversive procedures (Figure 2).

### 3.2. Long-Term Behavioral Effects of Flavonoids after Exposure to SPS

#### 3.2.1. Locomotor Activity Test

**Figure 3:**
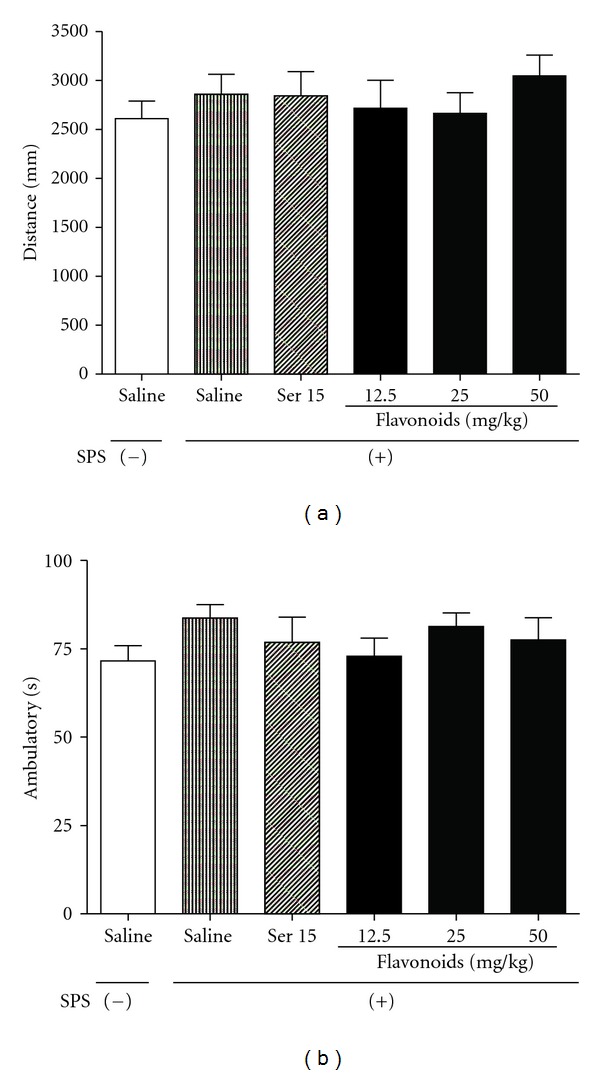
The effects of repeated treatment with Ser or flavonoids on locomotor activity in SPS-exposed rats. On day 13 after SPS procedure, the distance (a) and ambulatory time (b) were measured. Daily administration of Ser or flavonoids was started from the first day after the SPS procedure. Each column represents the means ± SEM (*n* = 10).

To examine the possibility that SPS and/or drug treatments influenced the baseline locomotor activity in “fear-conditioned” rats (subjected to fear conditioning 24 h before the measurement), we investigated the level of spontaneous locomotor activity for each group. The results showed that there was no significant difference between groups, indicating that neither SPS nor chronic drug treatments affected the spontaneous activity of rats (Figures 3(a) and 3(b)).

#### 3.2.2. Contextual Freezing

**Figure 4:**
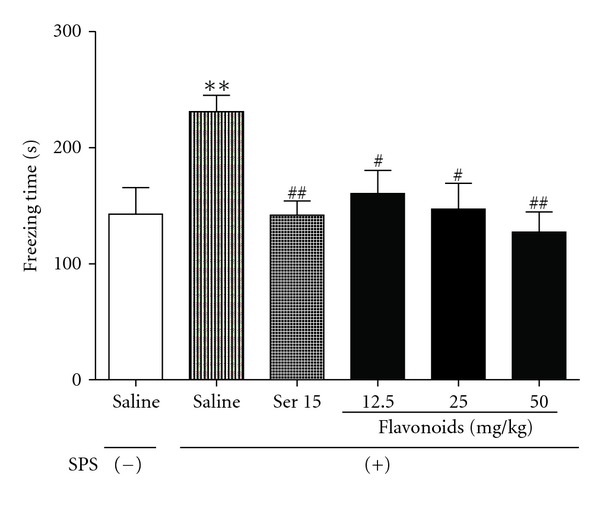
The effects of repeated treatment with Ser or flavonoids for 15 days on contextual freezing in SPS-exposed rats. The total time of freezing behavior was determined on day 15. Daily administrations of flavonoids were started from the first day after the SPS procedure. Data are presented as mean ± SEM (*n* = 10). **P < 0.01 compared with SPS (−); ^#^ *P* < 0.05, ^##^ *P* < 0.01 compared with SPS-exposed group (ANOVA followed by Dunnett's *t*-test).

One-way ANOVA analyses revealed that exposure to SPS, followed by a 14-day undisturbed period, significantly increased the contextual freezing response, compared to the control (*F*[5, 54] = 4.028, *P* < 0.01). A 14-day chronic coadministration with Ser alleviated the enhanced contextual freezing in rats experienced SPS (*P* < 0.01). Such effect was also observed with flavonoids (12.5, 25, and 50 mg/kg) treatment (Figure 4).

#### 3.2.3. EPM Test

**Figure 5:**
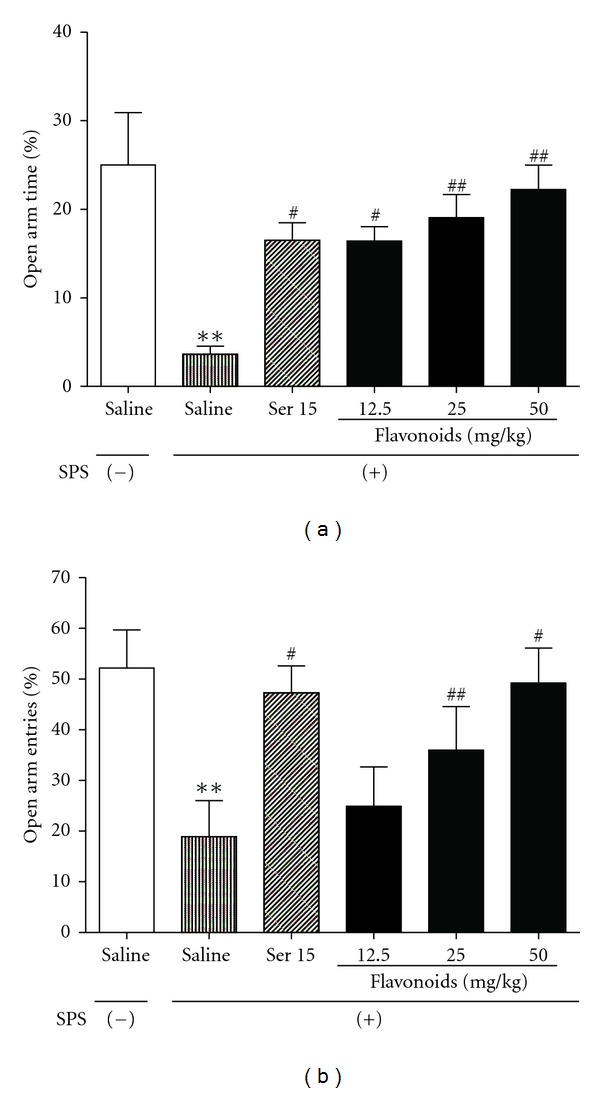
The effects of repeated treatment with Ser or flavonoids on SPS-exposed rats in EPM test. On day 18 after SPS procedure, percent time spent in (a) and numbers of entry into open arms (b) were recorded. Daily administrations of Ser or flavonoids were started from the first day after the SPS procedure. Data are expressed as mean ± SEM (*n* = 10). **P < 0.01 compared with SPS (−);^#^ *P* < 0.05, ^#  #^ *P* < 0.01 compared with SPS-exposed group (ANOVA followed by Dunnett's *t*-test).

As shown in Figure 5, one-way ANOVA analyses revealed that SPS-exposed animals showed significant reductions in percent time spent in open arms (*F*[5, 54] = 5.765, *P* < 0.01), and in percent number of entries into open arms (*F*[5, 54] = 3.863, *P* < 0.01). Post hoc comparisons further showed that chronic coadministration with Ser (15 mg/kg) significantly increased the above 2 declined parameters compared to control animals, and so did the repeated administrations of flavonoids (Figure 5).

### 3.3. Monoamine Neurotransmitters

The levels of NE, 5-HT, and DA in hippocampus and prefrontal cortex of rats were measured in the end of the EPM test. As shown in Table 1, in hippocampus, statistical analysis revealed that SPS significantly reduced 5-HT level (*F*[5, 54] = 7.546, *P* < 0.05), which was clearly reversed by the chronic administration of positive control Ser (*P* < 0.01); such effect was also mimicked by flavonoids treatment at 25 and 50 mg/kg, respectively. 

In addition, there was a trend of increase in NE level in hippocampus and prefrontal cortex, though with no statistical significance. A decreased tendency of NE level was also observed in these two brain regions with chronic flavonoids treatment. The levels of monoamine metabolites in hippocampus and prefrontal cortex of rats were also measured, but no statistically significant alteration was observed among groups. 

## 4. Discussion

Our results demonstrated that repeated situational reminders followed by electric foot shocks elicited the acquisition of conditioned fear, and the mice showed an innate aversive freezing behavior. A repeated flavonoids treatment significantly increased the time spent in the aversive-like context, indicating that flavonoids alleviated the fear feeling of the stressed animals to the context associated with the traumatic event. Moreover, the aversive procedure did not affect the animals' spontaneous locomotor activity (data not shown) and the number of climbed steps in the staircase test in mice. These results were in accordance with the studies of Pynoos et al. [33], who showed that foot-shocks associated to situational reminders did not affect the motor activity of male mice in an open field test performed 3–6 weeks after the first foot-shock. The present study indicated that the aversive foot-shocks followed by repeated reminders are a reliable long-lasting animal model for PTSD, and flavonoids showed a therapeutic effect in this animal model within certain dose range.

There are accumulating evidences showing that SPS rats exhibit symptoms of increased arousal, such as exaggerated fear responses to trauma-related and -unrelated stimuli [34, 35], which is consistent with the observation in PTSD patients who showed enhanced anxiety and fear in response to stimuli unrelated to trauma. The SPS procedure has been found to enhance contextual fear conditioning, and the freezing behavior may serve as a good assessment for the severity of anxiety due to hippocampal dysfunction [36, 37]. 

Data presented here demonstrated that contextual freezing was significantly enhanced in rats exposed to SPS, while chronic administration of flavonoids successfully reversed these adverse effects. EPM, a model using the natural fear of rodents to avoid open and elevated places, has been well validated in detecting responses to external stressful stimuli. It has been found that SPS procedure induced an anxiety-like behavior in the EPM test [38, 39]. The present study also showed that SPS exposure produced representative anxiety-like behavior, as evidenced by the fact that SPS-exposed animals significantly decreased their percentage of time spent in and number of entry into the open arms, while flavonoids reversed these behavioral changes and alleviated the anxiety in rats after SPS exposure. We also found that SPS and/or XBXT did not significantly influence the spontaneous locomotor activity in rats, suggesting that the behavioral changes observed in this study were not due to the change in basal locomotor activity. 

While brain 5-HT plays an important role in the etiology of PTSD, 5-HT systems are implicated in the treatment of PTSD too. Decreased serotonin levels in animal models and human studies have been associated with increased impulsivity, aggression, fear, and sadness/depression. Furthermore, the improvement of symptoms following treatment with serotonergic antidepressants (e.g., SSRIs) has been one explanation supporting the role of serotonergic dysfunction in PTSD [40]. The efficacies of SSRIs in treating PTSD symptoms are likely mediated by their enhancement of serotonergic function and subsequent improvement in modulation of anxiety, anger, mood, and impulsivity [41–44].

Numerous studies indicated that hippocampal serotonin dysfunction may be involved in enhanced contextual freezing in SPS rats [45, 46]. Our previous studies demonstrated that chronic flavonoids administration significantly increased hippocampal 5-HT and its metabolite 5-hydroxyindoleacetic acid levels in chronically stressed rats [26]. And we found that single flavonoids (25, 50, and 100 mg/kg, p.o.) administration significantly potentiated the mouse head-twitch response induced by 5-HTP. We also found that decrease of immobility time in mouse tail suspension test was completely prevented by p-chlorophenylalanine (PCPA, an inhibitor of serotonin synthesis) pretreatment. PCPA is an irreversible inhibitor of the enzyme tryptophan hydroxylase. It was reported that PCPA treatment at dose of 300 mg/kg for 3 consecutive days, which is the same as used in our previous study, produced partial but highly significant reductions (over 60%) in brain 5-HT level, while noradrenaline and dopamine levels were not affected. Results from these tests indicated that there was an indispensable involvement of the serotonergic system in the mechanism of flavonoids [25]. Using HPLC-ECD, our study also confirmed the possibility that the behavioral effects of flavonoids in SPS model were through increasing the 5-HT concentrations in hippocampus. Taken together, we speculate that the serotonergic action of flavonoids might be similar to that of SSRIs; however, further investigations on the presumptive adaptive changes such as hippocampal serotonin receptors are still needed to elucidate the precise role of flavonoids. 

It should be noted that, PTSD is a complex disorder that involves several different neurochemical and neuroendocrine alterations, and cannot be explained by a single system. Several papers reported that activation of GABA_A_-receptor resulted in various effects including: anxiolysis, cognitive effects, sedation, muscle relaxation, and anticonvulsant actions. These functions, particularly the first two would seem to have particular benefits for PTSD, yet short-term and long-term placebo-controlled studies have failed to demonstrate their efficacy. So whether GABA is involved in the anti-PTSD effect of flavonoids requires further study.

As a traditional Chinese herbal decoction comprising multiple ingredients, XBXT has also been studied for its chemical components. To date, 22 compounds, including 21 flavones, have been isolated from XBXT, and the rough HPLC fingerprint of the extract demonstrate that the major constituents of XBXT are flavones, flavonols, isoflavones, and their glycosides [25–27]. Extensive studies indicate that flavonoids act on different systems in the brain. Flavanols, flavanones, and anthocyanins may act in protective ways, increasing the cerebral blood flow and protecting the neurons from inflammatory processes-mediated cell injury. Flavonoids of several classes are inhibitors of MAO-A and -B, thus mediating an antidepressant or anti-Parkinson's activity. And also, flavonoids show potential to protect neurons from neurotoxins-induced injury. Furthermore, flavones may interact with the GABA_A_-receptor, producing sedation, anxiolytic, or anticonvulsive effect. Therefore, it seems plausible that beside the serotonergic activation, flavonoids may also exert its effect via other pathways, and the precise mechanisms of action warrants further studies, which now are undertaken by our group.

## 5. Conclusion

Over the past decade, herbal medicine has attracted increasing interest from the psychiatry research community for its better compliance and lower side effects. This is largely because of that many herbal preparations have been found to exert beneficial effects on various psychiatric conditions in experimental animal and clinical studies [18–22, 47]. Although the behavioral and transmitter changes observed in the current model confirm that flavonoids may be an effective herbal candidate to treat PTSD mainly through its serotonergic activation, further experiments are needed to clarify the exact molecular mechanisms underlying its effects and to better understand the neuropathological changes in PTSD.

## Figures and Tables

**Table 1:** The effects of repeated treatment with Ser or Flavonoids on the level of monoamine and its metabolite (ng/g tissue) in hippocampus and prefrontal cortex of rats subjected to SPS. Data are presented as the mean ± SEM, *n* = 4–6. *P < 0.05, compared with SPS (−); ^##^ *P* < 0.01 compared with SPS (ANOVA followed by Dunnett's *t*-test).

Groups	SPS (−)	SPS + saline	SPS + Ser (mg/kg)	SPS + Flavonoids (mg/kg)		
			15	12.5	25	50
Hippocampus						

5-HT	156.80 ± 5.42	118.20 ± 3.192*	199.21 ± 12.26^##^	153.81 ± 10.93	172.00 ± 13.79^##^	178.40 ± 9.90^##^
5-HIAA	456.20 ± 20.69	415.60 ± 36.16	343.60 ± 21.02^##^	465.60 ± 37.50	494.90 ± 38.21	484.40 ± 17.65
NE	155.90 ± 14.95	198.10 ± 23.71	182.70 ± 24.92	157.40 ± 7.77	187.50 ± 17.51	191.60 ± 22.08
DA	19.58 ± 3.20	22.94 ± 3.13	14.33 ± 0.99	15.49 ± 2.39	15.45 ± 1.33	17.46 ± 2.24
DOPAC	34.27 ± 7.69	32.22 ± 3.110	29.12 ± 2.91	26.35 ± 3.44	28.82 ± 2.18	30.12 ± 3.45

Prefrontal cortex						

5-HT	238.31 ± 11.47	257.45 ± 16.23	255.90 ± 39.32	233.20 ± 16.11	231.40 ± 15.89	264.31 ± 18.66
5-HIAA	371.82 ± 5.60	313.0 ± 45.29	458.50 ± 27.56^#^	402.90 ± 22.88	404.50 ± 20.44	445.50 ± 21.27^#^
NE	129.30 ± 16.21	155.0 ± 9.65	137.60 ± 17.86	124.80 ± 15.77	133.00 ± 11.24	146.80 ± 16.47
DA	48.09 ± 2.43	50.36 ± 8.22	49.87 ± 4.69	43.14 ± 3.76	36.25 ± 5.26	44.44 ± 0.98
DOPAC	33.23 ± 2.69	36.67 ± 2.77	39.88 ± 5.25	35.77 ± 2.66	33.57 ± 1.47	36.04 ± 2.89

## Abbreviations

ANOVA: Analysis of variance
AUC: Area under curve
DA: Dopamine
HPLC-ECD: High-performance liquid chromatography with electrochemical detection
5-HT: Serotonin
NE: Norepinephrine
PTSD: Posttraumatic stress disorder
SPS: Single prolonged stress
SSRIs: Selective serotonin reuptake inhibitors
TCM: Traditional Chinese medicine.
